# Psychometric properties and longitudinal validation of the self-reporting questionnaire (SRQ-20) in a Rwandan community setting: a validation study

**DOI:** 10.1186/1471-2288-11-116

**Published:** 2011-08-16

**Authors:** Willem F Scholte, Femke Verduin, Anouk van Lammeren, Theoneste Rutayisire, Astrid M Kamperman

**Affiliations:** 1 Department of Psychiatry, Academic Medical Center, University of Amsterdam, Netherlands, Meibergdreef 5, 1105 AZ Amsterdam, Netherlands; 2 Equator Foundation, Netherlands, Nienoord 5, 1112 XE Diemen, Netherlands; 3 Episcopal Church of Rwanda, Diocese of Byumba, Rwanda, P.O. Box 17, Byumba, Rwanda; 4 Department of Psychiatry, O3 Mental Health Care Research Center, Erasmus MC, Rotterdam, Netherlands, 's-Gravendijkwal 230, 3015 CE Rotterdam, Netherlands

## Abstract

**Background:**

This study took place to enable the measurement of the effects on mental health of a psychosocial intervention in Rwanda. It aimed to establish the capacities of the Self-Reporting Questionnaire (SRQ-20) to screen for mental disorder and to assess symptom change over time in a Rwandan community setting.

**Methods:**

The SRQ-20 was translated into Kinyarwanda in a process of forward and back-translation. SRQ-20 data were collected in a Rwandan setting on 418 respondents; a random subsample of 230 respondents was assessed a second time with a three month time interval. Internal reliability was tested using Cronbach's alpha. The optimal cut-off point was determined by calculating Receiver Operating Curves, using semi-structured clinical interviews as standard in a random subsample of 99 respondents. Subsequently, predictive value, likelihood ratio, and interrater agreement were calculated. The factor structure of the SRQ-20 was determined through exploratory factor analysis. Factorial invariance over time was tested in a multigroup confirmatory factor analysis.

**Results:**

The reliability of the SRQ-20 in women (α = 0.85) and men (α = 0.81) could be considered good. The instrument performed moderately well in detecting common mental disorders, with an area under the curve (AUC) of 0.76 for women and 0.74 for men. Cut-off scores were different for women (10) and men (8). Factor analysis yielded five factors, explaining 38% of the total variance. The factor structure proved to be time invariant.

**Conclusions:**

The SRQ-20 can be used as a screener to detect mental disorder in a Rwandan community setting, but cut-off scores need to be adjusted for women and men separately. The instrument also shows longitudinal factorial invariance, which is an important prerequisite for assessing changes in symptom severity. This is a significant finding as in non-western post-conflict settings the relevance of diagnostic categories is questionable. The use of the SRQ-20 can be considered an alternative option for measuring the effect of a psychosocial intervention on mental health.

**Trial registration:**

Nederlands Trial Register NTR1120.

## Background

### A psychosocial intervention in Rwanda

The country of Rwanda experienced extreme violence during a genocidal three months period starting in April 1994. Over the preceding years, the country's northern Gicumbi district had already been confronted with repeated acts of violence stemming from the same ethnic conflict. Although sixteen years have passed since, many inhabitants still suffer from the emotional sequelae.

Since early 2006 a psychosocial community intervention is carried out in the Northern Province in Rwanda, in and around a small city called Byumba. It is a therapeutic group intervention (sociotherapy) aiming at social bonding and mental health recovery [1-3]. No diagnostic criteria for participation have been defined, and the intervention is open to any adult (≥ 16 years) wanting to participate. Community members can also personally be invited when considered psychosocial problem cases by local sociotherapy group facilitators. Under the lead of these facilitators, sociotherapy groups meet weekly during a period of fifteen weeks. Every four months a new series of groups start. Groups contain adult participants of both sexes with a wide age distribution.

### Measurement of mental health in a post-conflict setting

When contemplating our choice for an instrument to measure the intervention's effect on mental health, we were aware of the questionnable relevance of diagnostic categories as defined by DSM-IV [4] or ICD-10 [5] in a population recently affected by systematic violence [6-8]. The high prevalence estimates of specific mental health problems usually established in such populations may reflect normal responses to severe environmental stress instead of disorders [9,10]. Additionally, the intervention studied does not focus on subjects suffering from any specific disorder. Instead, local community leaders' lay criteria for being a psychosocial problem case determine who will be beneficiaries of the intervention. Therefore, we chose to use a general case finding instrument rather than one or more instruments indicative of specific diagnoses. For this purpose we selected the Self-Reporting Questionnaire (SQR-20) [11,12].

### Validity of instrument translation

As the SRQ-20 had never been used in Rwanda before, we needed to translate the instrument into the country's local language, Kinyarwanda, and to establish its validity and optimal cut-off point. English-language research instruments must be carefully adapted and translated before use in another culture. Different terminologies exist to classify criteria such adaptations must meet [13]. Manson [14] discusses adaptation of instrument items in terms of comprehensibility (meaning of item is evident), acceptability (item is not offensive), relevance (item relates to the underlying construct) and completeness (item fully covers equivalents between cultures). When connecting these terms to the forms of equivalence between original and translated instruments as mentioned earlier by Flaherty et al. [15], comprehensibility relates to semantic equivalence, acceptability to technical equivalence, relevance to content equivalence, and completeness to semantic, criterion or conceptual equivalence [13].

### Longitudinal validity

We did not only use the SRQ-20 for its original purpose, i.e. case detection, but also to assess changes in scores over time. To our knowledge such use of the instrument has only been described once [16], but no data were provided about its longitudinal validity. We had to establish the instrument's capacity to meet additional psychometric criteria regarding its factorial solution. Changes in the score of a symptom checklist preferably reflect changes in the severity of the (possible) disorder. However, changes in test score may also reflect a reappraisal of the items, i.e. a reappraisal of the symptoms or a reappraisal of their impact. This makes changes in mean scores difficult to interpret. If the factorial solution of the instrument is stable over time, the latter sort of change can be ruled out since item loadings are affected by a reappraisal of the items [17]. Consequently, factorial invariance of the SRQ-20 is a prerequisite for assessing changes in symptom severity.

### Study objectives

The aim of this study was multiple. First we assessed the SRQ-20's capacity to screen for mental disorder in a Rwandan community setting. Next, we evaluated the psychometric properties of the instrument. Finally, we tested the stability of the factorial solution over time.

## Methods

### Ethics statement

Approval for this study was gained from the Medical Ethics Committee of the Academic Medical Center in Amsterdam, Netherlands.

### Study site and population

The sample for this study includes a mixture of beneficiaries of the intervention and their relatives, friends or close collaborators as well as individuals who were randomly selected in a nearby region not reached by the intervention.

### Instrument

The SQR-20 could be expected to show reliability and validity for case detection in the Rwandan context. The instrument is a twenty items subset of the SRQ developed by the World Health Organization for screening the presence of mental disorder in patients contacting primary health care settings [11,18]. The complete SRQ consists of twenty-five questions, which have to be answered by 'yes' or 'no'. Of these twenty-five questions, twenty are related to neurotic symptoms, four to psychotic symptoms and one to convulsions. The SRQ-20 consists of the neurotic items only. These reflect depressive symptoms, anxiety, and psychosomatic complaints and have been found to detect probable cases of common mental disorder with reasonable accuracy [18].

The instrument met several criteria for use in this study, which was carried out by interviewers with limited training, within a limited period of time, among a large number of respondents, most of whom were illiterate. It is a self-report questionnaire; for illiterate respondents the questions may be read aloud by interviewers. Its administration time is 5-10 minutes. The questions of the instrument are written in a simple, easy to understand language, and cover many important areas of psychopathology. The SRQ-20 has been used in many community-based surveys conducted in developing countries [11,19-21]. Additional to the widespread use of the 20 items version, the decision not to include items related to psychosis for the present study was also based on information from sociotherapy group facilitators: psychotic persons tended not to participate in the groups; not any psychotic participant was known of.

The SRQ-20 items are scored 0 ('no', symptom absent) or 1 ('yes', symptom present). Item scores are summarized to obtain a total score. A score above cut-off point indicates the existence of a probable menatl disorder. A cut-off score of 8 is widely used. However, optimal cut-off scores are shown to vary considerably across cultures, languages, settings, gender et cetera [21-28]. Factor structures of the SRQ-20 also vary across populations, ranging from two to seven [21,29,30]. Overall correspondence among the factor structures of the SRQ-20 is not found. So, at this stage the use of factor structures as subscales is not recommended. Besides, the variation in cut-off scores and factor structures emphasizes the need for performing separate validity studies among different populations.

### Instrument translation

For this study, all SRQ-20 items were translated into Kinyarwanda by a bilingual Rwandan collaborator of the evaluation study (ThR), familiar with the intervention program and the (mental) health issues addressed by the instrument. Blind back-translation was done by another bilingual Rwandan, who was independent from, and not familiar with the intervention program or the study. This back-translation was examined by the first translator together with two other researchers (WFS, FV), which led to one minor change in the translation. Subsequently, the comprehensibility, acceptability, relevance and completeness of all items were discussed with the eight Rwandan interviewers working for the evaluation study. No changes were considered necessary [3].

### Study sample

The SRQ-20 was administered to a sample of 418 adults (16 years and up); amongst these were 97 intervention (sociotherapy) group participants who had been selected randomly out of ten sociotherapy groups in or relatively near the study site (Byumba city) and yet correctly representing the intervention group with respect to gender and to urban or rural living situation; 92 relatives, friends or close collaborators of intervention group participants, i.e. one such person for every participant (these persons had each been randomly selected out of five persons listed by every group participant); and 229 randomly chosen other inhabitants of the district (the effectiveness study's control group). This sample will be referred to as the baseline sample (BA). Informed consent was obtained by use of an explanatory text, which because of the high illiteracy rate was read aloud. The interviewers were four men and four women who had been trained by two of the researchers (WFS, FV) during a three day training. They were all Rwandan sociology students at the 'Institut Polytechnique de Byumba'. They administered the questionnaire in a respectful way, not stopping respondents at 'yes' or 'no' and allowing more lengthy explanations.

A subsample of 99 was formed to establish the instrument's local validity and optimal cut-off point. This subsample will be referred to as the clinical interview (CI) sample. As no diagnostic or screening instrument with proven validity in a Rwandan context exists to be used as a 'gold standard', this subsample was clinically assessed by experienced clinicians. Meetings of intervention group participants and their selected relatives, friends or close collaborators were organized for administration of the SRQ-20. All respondents scoring 8 or above and an equal number scoring 7 or below, irrespective of whether they were intervention group participants or not, were also assessed by the clinicians, who were blind for the SRQ-20 scores. This procedure was repeated over similar meetings with different respondents untill 99 clinical assessments were completed.

Unfortunately, these assessments could not be done by trained clinicians from the same culture as the respondents. Rwanda harbours only three native psychiatrists, and these were not available for the study. Instead, the assessments were carried out by three of the Dutch researchers (WFS, FV, AvL) who are clinicians as well; all have broad experience in assessing psychopathology in non-western populations. They conducted a semi-structured interview covering listed core symptoms of the disorders that were most likely to prevail in the specific context, i.e. depression, posttraumatic stress disorder, generalized anxiety disorder and panic disorder. It also contained questions to exclude psychosis and normal grief. If an assessor concluded that any of the mentioned disorders was present or possibly present in a subject, this subject was recorded as a 'case'. Interview agreement among the assessors was obtained by the following procedure: The assessors themselves designed the list of core symptoms as mentioned above. Next, two assessors witnessed an assessment carried out by the third one, after which all three independently made their own conclusions about 'caseness' of the respondent. There wasn't any verbal or non-verbal communication between the three clinicians untill all three had separately made and documented their diagnostic conclusions. The assessors took turns in taking the witnessing or the interviewing role. This procedure was followed sixteen times, fifteen times of which all three assessors drew identical conclusions. This resulted in 96% overall agreement and an interrater reliability (Fleiss' kappa) of 0.92. Since the assessors did not each by themselves conduct assessments of the same subjects, these agreement statistics might be inflated. The assessments were carried out with the aid of two of the intervention program's translators, familiar with western as well as local psychological idioms, and aware of the relevance of literal and neutral translation. The translators were also quite familiar with the clinicians, which allowed them to note and clarify possible (cross-cultural) misunderstandings between interviewers and respondents.

To assess the instrument's longitudinal validity a second, larger subsample (230 respondents) was re-assessed with the SRQ-20 by the same interviewers after a three months period, i.e. right after the intervention. This subsample will be referred to as the re-assessment (RA) sample. It was formed by all sociotherapy group participants and their relatives, friends or close collaborators who were available for both assessments, completed by a random selection of respondents from the control group matched on sex and age.

### Data analysis

T-tests were conducted to compare mean scores of the SRQ-20. Internal reliability of the instrument was analyzed with Cronbach's alpha. Receiver operating characteristics (ROC) curves were used to explore the overall accuracy of the instrument to distinguish correctly between case and non-case, characterized by an area under the curve value (AUC). AUC values range from 0 to 1.0, in which a value of 1.0 indicates a perfect prediction and a value of 0.5 indicates a prediction equal to chance. It was tested whether the criterion value of the SRQ-20 exceeded chance level (AUC > 0.5). Diagnostic sensitivity is the probability of a positive test result given the condition is present. Specificity is the probability of a negative test result given the condition is absent. Subsequently, a positive predictive value (PPV) is the probability of a positive diagnosis after a positive screening, and negative predictive value (NPV) is the probability of a negative diagnosis after a negative screening [31]. Predictive values range from 0 to 1, in which a value closer to 1 reflects a better predictive value. Positive likelihood ratio (PLR) and negative likelihood ratio (NLR) provide direct estimates of an individual's chance of caseness. Likelihood ratios incorporate both sensitivity and specificity of the test. The PLR indicates how much the odds of the disease increase when a test is positive. The NLR indicates how much the odds of the disease decrease when a test is negative [32].

The degree of agreement between the results from the SRQ-20 and the clinical interviews is expressed both in percentage agreement and in Cohen's kappa coefficients. A kappa value of 1 reflects a perfect agreement between both observators, a kappa value of 0 reflects a degree of agreement as expected on base of chance. Kappa values in the range of 0.4 - 0.75 can be interpreted as fair, kappa values exceeding 0.75 as good, and kappa values below 0.2 as slight agreement [33]. Diagnostic sensitivity and diagnostic specificity were plotted against each other to establish the optimal cut-off point. ROC-analysis and assessment of the psychometric qualities of the SRQ-20 were performed in the CI sample.

To test the longitudinal factorial invariance of the SRQ-20 we used exploratory factor analyses and confirmatory factor analyses. Product-moment correlation coefficients matrix was used to perform the factor analyses. We started with performing an exploratory factor analysis using principal axis factoring extraction in the BA sample to uncover the covariances between the twenty items of the SRQ-20. To facilitate interpretation varimax rotations were performed on the initial factor solutions. Based on the Kaiser-Guttman rule, factors with eigenvalues larger than 1 were retained for subsequent analyses. An item was assigned to a factor and used for factor labelling if its loading on that respective factor was larger than 0.35 and its loading to any other factor smaller than 0.35 [34]. Subsequently, confirmatory factor analyses were performed.

Confirmatory factor analysis (CFA) involves testing a series of hypothesized models relating to the instrument's measurement properties across samples. We started with testing the absolute fit of the factor structure in the BA and RA sample, consecutively. Subsequently, we used multigroup CFA to examine the extent of measurement invariance across these samples. In this analysis the fit between two hypothesized factor models is compared [35]. We distinguish the following models: 1) Model A: a model in which the number and pattern of factors are equal across samples; 2) Model B: model A with the additional constraint that factor loadings are equal across samples; 3) Model C: model B with the additional constraint that covariance matrices of factors are equal across samples; 4) Model D: model C with the additional constraint that error variances are equal across samples. Increment of fit between 1) model A and model B; 2) model B and model C; 3) model C and model D was tested using a *χ^2 ^*test. If *Δχ^2 ^*is not significant, the hypothesis of factorial invariance is tenable [36]. Sample sizes were adequate to test the fit of medium sized models [37]. Datanalyses were performed using PASW 17.0. Confirmative factor analyses were performed using Amos 16.0 [38].

## Results

### Sociodemographic characteristics

Male-female ratios were around 2:3 for the BA and CI samples and around 1:3 for the RA sample. Mean ages were around 35 years for the BA and CI samples and around 37 years for the RA sample. See Table 1.

**Table 1:** Sociodemopgraphic characteristics of the three study samples

	Baseline (BA) sample	Clinical Interview (CI) sample	Re-assessment (RA) sample
**Number of respondents**	418	99	230
**Sex**			
Males, no (%)	163 (39, 0)	42 (42, 4)	55 (23, 9)*
Females, no (%)	255 (61, 0)	57 (57, 6)	175 (76, 1)
**Age **(mean, S.D.)	16-87 (*μ *= 35, 3; S.D. = 15, 0)	16-74 (*μ *= 34, 7; S.D. = 14, 1)	16-77 (*μ *= 37, 2; S.D. = 13, 9)*
**Treatment condition^1^**			
Direct, no (%)	97 (54, 8)	52 (52, 5)*	90 (39, 1)*
Indirect, no (%)	92 (23, 2)	47 (47, 5)	77 (33, 5)
Control, no (%)	229 (22, 0)	-	63 (27, 4)

* *P *< 0.05, means and proportions tested against the total sample

^1 ^direct = sociotherapy group participants; indirect = relatives, friends or close collaborators of sociotherapy group participants; control = random community sample.

### Predictive validity

Reliability of the SRQ-20 over all samples is considered good (alphas ranging from 0.83 in CI sample to 0.87 in BA sample). Reliability in men and women, respectively, was also considered good (men: *α *= 0.81; women: *α *= 0.85). Mean total score of the SRQ-20 is 8.5 (S.D. = 3.5). Total scores showed no significant differences between men and women. Persons diagnosed having a mental disorder by the clinicians scored significantly (T(97) = 4.325; p < 0.00) higher (mean = 11.3; S.D. = 4.1) compared to those having no mental disorder (mean = 7.3; S.D. = 4.2). As no cases of psychosis were identified during clinical interviews, the existence of psychosis can be ruled out as a cause for disagreement between SRQ-20 scores and clinical diagnoses.

**Figure 1:**
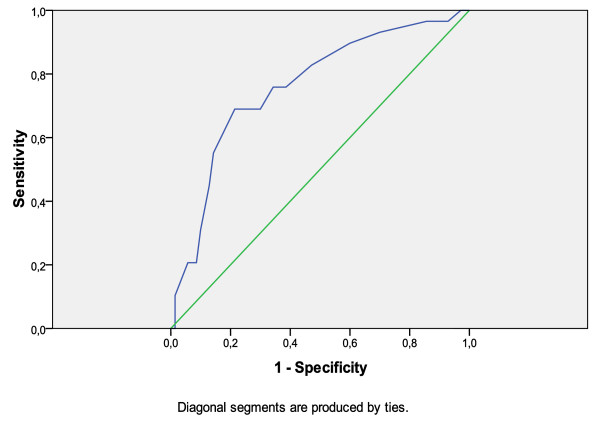
**Receiving Operator Characteristic (ROC) curve of the SRQ-20 scores in the presence or absence of caseness as diagnosed by the clinicians**.

The SRQ-20 performed moderately well in detecting common mental disorders. The AUC was 0.76. When analysed separately for men and women the SRQ-20 showed to perform equally well in men (AUC = 0.74) and women (AUC = 0.76). See Figure 1 and Table 2.

**Table 2:** SRQ-20: Area under the curve, lower and upper limit

	AUC	LL	UL	*Z*-value	*P*-value
**Male**	0.74	0.59	0.90	3.077	0.013
**Female**	0.76	0.62	0.91	3.467	0.002
**Total**	0.76	0.66	0.86	4.815	< 0.001

In evaluating the SRQ-20 as a potential screener for common mental disorder the most appropriate cut-off score is a trade-off between a high sensitivity and an acceptable specificity. In Rwanda, the SRQ-20 performs moderately well as a screener with a score of 10 as the optimal local cut-off point (sensitivity 0.69; specificity 0.79; see Table 3). The SRQ-20 performed better in women than in men. Cut-off scores differed also between men and women. The optimal cut-off point for men is 8 (sensitivity 0.69; specificity 0.65), while the optimal cut-off point for women is 10 (sensitivity 0.81; specificity 0.80). In evaluating likelihood ratios the optimal cut-off score combines the largest PLR with the smallest NLR. Both positive and negative predictive values and positive and negative likelihood ratios confirm the optimal cut-off scores. The PPV's of the cut-off scores can be considered good. The NPV's are relatively poor. This is consistent with the assumption that common mental disorder is prevalent in this traumatized society.

**Table 3:** Psychometric properties of the SRQ-20 with different cut-off scores

	Cut-off	Sensitivity	Specificity	PPV	NPV	PLR	NLR	Agreement	kappa
**Male**	7	0.69	0.59	0.81	0.43	1.67	0.52	62%	0.24
	8*	0.69	0.65	0.83	0.47	2.01	0.47	67%	0.31
	9	0.54	0.69	0.77	0.44	1.74	0.67	64%	0.21
**Female**	8	0.81	0.66	0.90	0.48	2.38	0.28	70%	0.39
	9	0.81	0.71	0.91	0.52	2.78	0.27	74%	0.44
	10*	0.81	0.80	0.92	0.62	4.16	0.23	81%	0.56
	11	0.69	0.83	0.87	0.61	4.03	0.38	79%	0.50
**Total**	7	0.76	0.61	0.86	0.45	1.97	0.39	66%	0.31
	8	0.76	0.66	0.87	0.48	2.12	0.37	69%	0.36
	9	0.69	0.70	0.84	0.49	2.30	0.44	70%	0.35
	10*	0.69	0.79	0.86	0.57	3.22	0.39	76%	0.45
	11	0.55	0.86	0.82	0.62	3.86	0.52	77%	0.42

* Optimal cut-of score; scores *above *this cut-off value give the best indication for possible psychopathology.

Note: PPV: positive predictive value; NPV: negative predictive value; PLR: positive likelihood ratio; NLR: negative likelihood ratio.

Cohen's kappa values of the SRQ-20 for the optimal cut-off scores were found fair in the total sample and among the women. The kappa value was poor to moderate for men.

### Exploratory factor analysis

Principal factors extraction with varimax rotation was performed on all items of the SRQ-20 for the total sample (*n *= 418). Five factors were extracted, explaining 38% of the total variance. The number of items included for all five factors was 14. Six SRQ-20 items were not assigned to any factor, due to factor loadings < 0.35 (items 4, 5, 14 and 17) or factor loadings > 0.35 on multiple factors (items 6 and 8). The factors reflected the following content: factor 1: emotional and bodily symptoms of depression (items 1, 2, 3, 9 and 10); factor 2: disability (items 11, 12 and 13); factor 3: digestive complaints (items 18 and 20); factor 4: lack of energy (items 7 and 19); and factor 5: loss of self esteem (items 15 and 16). Eigenvalues ranged from 5.80 to 1.02.

### Confirmatory factor analysis

To determine whether the SRQ-20 factor structure was invariant over time, single and multi sample confirmatory factor analysis was performed on the five factors of the SRQ-20. The five factors were hypothesized to covary with one another.

The assumption of normality was evaluated through AMOS 16.0. The RA sample showed significant skewness. Mardia's coefficient for multivariate kutosis was 9,289 in the BA sample and 17,122 in the RA sample, indicating a non-normal multivariate distribution of the data. No outliers were observed (using Mahalanobis distance). CFA was performed using data from the BA sample (*n *= 418) and RA sample (*n *= 230). There were no missing data.

We identified the fit of the hypothesized model in the single samples. In the BA sample the hypothesized model showed good fit with the data, where *χ^2^*(67) = 93.243, RMSEA = 0.031, RMR = 0.008, GFI = 0.97, CFI = 0.98 and TLI = 0.97. In the RA sample the model showed excellent fit with the data, where *χ^2^*(67) = 70.001, RMSEA = 0.014, RMR = 0.008, GFI = 0.96, CFI = 1.00 and TLI = 0.99. Subsequently we conducted a multisample CFA. Maximum likelihood estimation was employed to test the fit of all models. The hypothesized model fitted the data well (*χ^2^*(134) = 163.27, *P *= 0.043). A follow-up Bollen-Stine bootstrapped analysis was performed with 200 replications to correct for non-normality of the data. This resulted in better *χ^2 ^*and *P *values, *χ^2 ^*= 146.36 and *P *= 0.214.

No significant differences were found between the unconstrained model and the models with constrained factors, factor loadings and covariances. There was, however, a significant difference between model C and model D, indicating a longitudinal invariance on the residual level. See Table 4 for detailed multigroup comparison fit indices.

**Table 4:** Results of multigroup confirmative factor analysis using data from BA sample (*n *= 418) and RA sample (*n *= 230)

	*χ^2^*	*Δχ^2^*	*Δ*df	*P*	RMSEA (90% CI)	RMR	GFI	CFI	TLI
**Model A:** **Equal factors**	163.27	-	-	-	0.018 (0.003-0.028)	0.008	0.97	0.99	0.98
**Model B:** **Equal factor loadings**	165.76	2.50	9	0.98	0.016 (0.000-0.025)	0.008	0.97	0.99	0.99
**Model C:** **Equal covariances**	190.29	24.53	15	0.06	0.018 (0.004-0.026)	0.012	0.96	0.98	0.98
**Model D:** **Equal error variances**	269.23	78.94	14	0.000	0.030 (0.023-0.036)	0.013	0.95	0.95	0.95

Note: *Δχ^2:^*: nested *χ^2 ^*difference; *Δ*df: nested df difference; *P*: *P*-value assuming the less restrained model to be correct; RMSEA: root mean square error of approximation; 90% CI: 90% confidence interval; RMR: root mean square residual; GFI: goodness of fit index; CFI: comparative fit index; TLI: Tuckler-Lewis Index.

## Discussion

This study shows the SRQ-20 can be used as a screener to detect mental disorder in a Rwandan community sample. However, cut-off scores need to be adjusted. Rwandan women and men have different optimal cut-off scores. Among men a cut-off score of 8 was optimal, among women a cut-off score of 10 was optimal. Differences between men and women were also found in a validation study among a traumatized population in Eastern Afghanistan [21]. Compared with other traumatized populations, the cut-off in the Rwandan population is relatively low, suggesting a more introvert expression of psychological distress.

In the present study the SRQ-20 performed less well in males than in females. This may be due to the country's atmosphere, which is still paranoid after the mass violence that took place. Especially men show a tendency not to trust others easily and to keep problems inside. Qualitative information consistently points out that men in Rwanda generally do not share emotional problems. This may have impacted the intervention's effect on men as well as the validity of data collected from male respondents. It may also explain the difference in optimal cut-off points between men and women.

A comparable problem may apply to ethnic background. Given the country's recent history this is an extremely sensitive issue, not to be addressed by interviewers during a brief one-time meeting. Besides, many residents have a mixed Hutu-Tutsi background - the distinction which is usually made. Yet, one can not rule out the possibility that ethnic background impacted the way people responded to the intervention as well as to certain questions of the interview, and therefore acts as an independent variable. The same goes for age, as age impacts the mental health consequences of past experiences. Our study samples represent all age groups, and therefore our findings only apply to the use of the SRQ-20 in random community samples.

For the present study we used a stratified sample to ensure sufficient variance in our SRQ scores. The actual prevalance of psychopathology in the Rwandan community is unknown and, as a result, we were not able to weigh the sample accordingly in our analyses. This is an important limitation of our study. Misrepresentation of the prevalence rate does not affect PPV and NPV estimations, but it does bias sensitivity, specificity, PLR, NLR, agreement and kappa estimations. However, the extent of this bias is unknown. Several studies in war affected populations and particularly in Rwanda found extremely high rates of depression, PTSD and other anxiety disorders [39-44]. The prevalence in our sample was 69%. It should be noted that we did not use diagnostic instruments but a screener, thereby identifying *possible *cases and capturing a *variety *of possible diagnoses in one measure. Therefore, a very high prevalence could be expected in our sample, quite possibly representing the actual prevalence. Future research is needed to confirm the sensitivity and specificity estimates of the SRQ-20 in a Rwandan community sample.

When dichotomous items are concerned, ideally a tetrachoric matrix should form the input of the exploratory factor analysis and confirmatory factor analyses [45]. In our case, the resulting tetrachoric matrix calculated using SPSS-Macro TetCorr Version 2.3 [46] from our data was non-positive definite, which made it impossible to compute a factor solution using principal axis factoring. This is a common problem in analysing symptom data, often caused by large correlations, insufficient sample size, or a non-normal underlying distribution [45,47]. Principal component analysis is less sensitive to these issues, but since our purpose was to structure the correlations among our variables, e.g., to explore a parsimonious representation among our measured variables instead of reducing the number of items, we decided against it. Also, the bias caused by using this method of analysis is less important when exploring the clustering of the items is the main purpose of the analysis, as it was in our case. All things considered, we chose to employ a less sophisticated method of analysis, i.c. employing a Pearsons correlation matrix as basis for our factor analysis. Previous studies on the factor structure of the SRQ also used a Person correlation matrix as basis of their analyses [21,29,30].

Since the factor structure of the SRQ-20 in this study proves to be time invariant, the instrument meets an important criterion to measure symptom change over time [17]. The results show that the number of factors, factor loadings and covariances of the factors remain equal over time. Only at residual level time invariances exist. This residual invariance might account for the differences in factor structures found across cultures. It is, however, important to realise that differences exist between our BA and RA samples, which may have caused residual invariance. Also, residual variances are reflective of individual variances in response to factors such as the intervention [48]. That is, at pretest the variances will be more homogeneous because individuals are more similar with regard to their level of psychological complaints. At posttest individual differences will be more pronounced because some participants respond favourably to the intervention, whereas others do not. Furthermore, in our samples data were not multivariate normally distributed. It is known that non-normality inflates the *χ^2^*-statistic of overall model fit, thereby increasing the chance of type 1 errors, i.e. the chance to abusively reject a hypothesized model [49]. Overall, methodologists agree that the test of equal residual variances is highly stringent and will rarely hold in realistic datasets [50]. Residual invariance is therefore not as important to the evaluation of measurement invariance as the test of equal form and factor loadings.

The data in our samples were categorical and non-normal in nature. Maximum likelihood estimation (MLE) of models with this type of data is not recommended. Asymptotic Distribution Free (ADF) or robust weighted least square estimator (WLSMW) might be a more appropriate choice. Unfortunately, the use of ADF requires sample sizes that exceed 1000 cases and small models. So, while ADF analysis may be theoretically optimal, it is not a practical method [51]. Also, WLSMW is not offered in AMOS 16.0. Since MLE is less problematic when analyzing the covariance matrix, and since Fouladi [52] found in a simulation study that the Bollen-Stine test of overall model fit performed well relative to other methods of testing model fit, we decided to estimate the model with MLE and additional Bollen-Stine bootstrapping.

The factor structure of the SRQ-20 in this study differed from factor structures reported in literature. Even in comparable settings numbers and contents of factors showed differences. For instance, in a primary care sample in an Eastern Afghanistan post-conflict setting, the SRQ-20 resulted in two factors, namely 'common disorder' and 'social disability' [21]. This study further emphasizes the need to establish the optimal cut-off scores for each setting, and not to use factors as separate subscales.

Lastly, this study confronted the researchers with various cross-cultural challenges. Extensive qualitative research and ample time and human resources are required to optimally deal with issues like the local validity of items of the SRQ-20, limited response options ('yes' or 'no'), and culturally sensitive clinical assessment. Our limited resources to tackle these issues may have impacted the cross-cultural validity of our findings.

## Conclusions

The SRQ-20 can be used as a screener to detect mental disorder in a Rwandan community sample. However, cut-off scores need to be adjusted. In this study setting, the instrument also shows longitudinal factorial invariance, which is an important prerequisite for assessing changes in symptom severity. This is a significant finding as in non-western post-conflict settings the relevance of diagnostic categories is questionable. The use of the SRQ-20 can be considered an alternative option for measuring the effect of a psychosocial intervention on mental health.

## Competing interests

The authors declare that they have no competing interests.

## Authors' contributions

WFS participated in conceiving and designing the study and in the data collection, and in drafting and editing the manuscript. FV participated in conceiving and designing the study and in the data collection, and in editing the manuscript. AvL participated in the data collection and in editing the manuscript. TR participated in the data collection and editing the manuscript. AMK participated in conceiving and designing the study, performed the statistical analysis and participated in drafting and editing the manuscript. All authors read and approved the final manuscript.

## Pre-publication history

The pre-publication history for this paper can be accessed here:

http://www.biomedcentral.com/1471-2288/11/116/prepub
